# Improvement of gastrointestinal discomfort and inflammatory status by a synbiotic in middle-aged adults: a double-blind randomized placebo-controlled trial

**DOI:** 10.1038/s41598-020-80947-1

**Published:** 2021-01-29

**Authors:** Audrey M. Neyrinck, Julie Rodriguez, Bernard Taminiau, Camille Amadieu, Florent Herpin, François-André Allaert, Patrice D. Cani, Georges Daube, Laure B. Bindels, Nathalie M. Delzenne

**Affiliations:** 1 Metabolism and Nutrition Research Group, Louvain Drug Research Institute, UCLouvain, Université catholique de Louvain, Avenue E. Mounier, Box B1.73.11, 1200 Brussels, Belgium; 2 Fundamental and Applied Research for Animal and Health (FARAH), Faculty of Veterinary Medicine, University of Liège, 4000 Liège, Belgium; 3 CEN Nutriment, CEN Group, Dijon, France; 4 WELBIO-Walloon Excellence in Life Sciences and BIOtechnology, UCLouvain, Université catholique de Louvain, Brussels, Belgium

**Keywords:** Microbial communities, Ageing, Randomized controlled trials, Gastroenterology

## Abstract

Several studies suggest that microbial alterations (dysbiosis) are intimately linked to chronic inflammation occurring upon aging. The aim of this study was to investigate the potential interest of a synbiotic approach (co-administration of a probiotic bacteria and a prebiotic dietary fibre) to improve gastrointestinal wellness and inflammatory markers in middle-aged people. Middle-aged subjects were randomized to take synbiotic (*Bifidobacterium animalis lactis* and fructo-oligosaccharides (FOS)) or placebo for 30 days. Stool frequency and consistency were improved in both placebo and synbiotic-treated volunteers while the synbiotic treatment significantly decreased the number of days with abdominal discomfort. Synbiotic treatment had no impact on mood dimensions, quality of life scores or the overall composition of the gut microbiota (16S rRNA gene sequencing of DNA extracted from stool). Importantly, plasma proinflammatory cytokines (interleukin (IL)-6, IL-8, IL-17a and interferon-gamma (IFNγ)) were significantly lower after 30 days of synbiotic supplementation. This effect appears to be independent of the gut barrier function. This study demonstrates that a combination of *B. animalis lactis* and the well-known prebiotic FOS could be a promising synbiotic strategy to decrease inflammatory status with improvement of gut disorders in middle-aged people.

## Introduction

The absolute number and the proportion of older people is increasing in most countries, with a continued rise in life expectancy^1^. Population ageing is particularly rapid within the European Union, due to low fertility rates and decreasing old age mortality^2^. Several health problems can be present or aggravated with ageing, which can compromise the quality of life. It is the case of constipation, one of the most frequent gastrointestinal disorders encountered in clinical practice in Western countries^3^. Importantly, chronic constipation is associated with significant impairment to quality of life^4^, represents a burden to healthcare delivery systems and results in large individual healthcare costs^5,6^. Constipation prevalence increases with age in women versus men^3,7^. Although laxatives are effective and commonly used in most patients, several side effects can often occur, especially with chronic use, which limits their use in the elderly^8^.

The availability of new, effective and safe treatments for chronic constipation without side effects would improve quality of life and the health status of the elderly.

Aging is also associated with a disruption of the immune system, including the release of several pro-inflammatory markers into the bloodstream, a phenomenon that has been called “inflammaging”. Indeed, many studies show that circulating concentrations of many inflammatory mediators are higher in the elderly than in young adults^1,9^. It is important to note that a previous report showed that subclinical elevations of inflammatory markers such as interleukin-6 (IL-6) are linked to the development of diabetes in middle-aged adults, demonstrating that low-grade systemic inflammation precedes and predicts the development of diabetes in adults^10^. Different tissues (adipose tissue, muscle), organs (liver and brain), systems (immune system) and ecosystems (intestinal microbiota) may contribute to the systemic state of low-grade inflammation observed during aging by altering the production of pro-inflammatory and/or anti-inflammatory mediators^1^.

The gut is populated by trillions of bacteria, archaea, eukarya, and viruses, constituting the gut microbiota. Five major microbial phyla (Firmicutes, Bacteroidetes, Proteobacteria, Verrucomicrobia and Actinobacteria) account for 98% of the intestinal microbiota^11,12^. Microbiota composition was a strong co-variate of measures of comorbidity, nutritional status, and markers of inflammation and microbial dysbiosis has been recently proposed as an additional hallmark and biomarker of aging^13–15^. Whether changes in gut microbiota are a cause or a consequence of aging in itself remains an exciting and challenging question. What has been demonstrated is that some gut bacteria promote aging-associated inflammation and that reversing microbiota dysbiosis represents an interesting therapeutic approach to reduce inflammation^9^. Microbiota-driven therapies, such as intake of probiotics, prebiotics or synbiotics, seem a promising approach to control inflammation increasing with age. Probiotics are specific microorganisms which have a beneficial effect on host health^16^ whereas prebiotics are nutrients selectively utilized by specific gut bacteria that confer health benefits to the host^17,18^. The administration of probiotics or prebiotics has been proposed as an innovative and valid alternative for functional or chronic constipation compared to traditional treatments with drugs^3,19–21^. The combination of selected probiotics and prebiotics can fit lead to the concept of “synbiotics”—which has been proposed as innovative therapeutic approach. A panel of nutritionists, physiologists and microbiologists recently updated the definition of a synbiotic to “a mixture comprising live microorganisms and substrate(s) selectively utilized by host microorganisms that confers a health benefit on the host”^22^. Although previous animal studies have demonstrated that microbiota-driven therapy changed the composition of the gut microbiota and decreased inflammatory markers^23^, whether this benefit can be translated to human remains controversial.

In the present study, we investigated the effects of a synbiotic-combining fructo-oligosaccharides (FOS) and *Bifidobacterium animalis lactis*—as an emerging strategy to improve intestinal transit and to relieve the symptoms of constipation in middle-aged people. In addition, we explored the gut microbiota, the quality of life and the inflammatory status as secondary endpoints.

## Results

### Baseline characteristics

Twenty-seven subjects (n = 27) were randomized for the study: 14 in the placebo group and 13 in the synbiotic group. The average age was 58 ± 6 years. The majority of the subjects (85%) included in this study were women. At baseline, the groups were similar in terms of age, sex, weight, blood pressure, serum transaminases, triglyceridemia, cholesterolemia and glycemia (Table 1). The compliance with consuming the study product was 94.9% and 96.7% for placebo and synbiotic, respectively. None presented major deviations from the protocol and all participated until the end of the study. Under these circumstances, the per protocol (PP) and intention to treat (ITT) results of the study were identical as they related to the same population.

**Table 1:** Baseline characteristics of participants.

	Placebo (n = 14)	Synbiotic (n = 13)	All (n = 27)
Women/men N (%)	12/2 (86/14)	11/2 (85/15)	23/4 (85/14)
Age (years old)	58 ± 7	58 ± 5	58 ± 6
Body weight (kg)	64.5 ± 11.4	69.1 ± 10.9	66.7 ± 11.2
BMI (kg/m^2^)	23.8 ± 3.2	24.7 ± 3.4	24.3 ± 3.3
SBP (mm Hg)	118.2 ± 15.8	118.2 ± 18.3	118.5 ± 16.7
DBP (mm Hg)	73.6 ± 6.6	75.8 ± 9.1	74.6 ± 7.8
ALAT (UI/l)	19.4 ± 6.1	24.8 ± 8.6	22.0 ± 7.7
ASAT (UI/l)	21.6 ± 4.2	24.1 ± 5.5	22.8 ± 4.9
Glycemia (g/l)	0.93 ± 0.10	0.97 ± 0.10	0.95 ± 0.10
Triglyceridemia (g/l)	0.96 ± 0.37	0.98 ± 0.43	0.97 ± 0.39
Cholesterolemia (g/l)	2.35 ± 0.40	2.38 ± 0.38	2.37 ± 0.39

Values are means ± SD. Baseline data were analyzed by Mann–Whitney test for continuous variables (p > 0.05) and Fisher test for categorical variables (ratio women/men; p > 0.05).

*ALAT* alanine aminotransferase, *ASAT* aspartate aminotransferase, *BMI* body mass index, *DBP* diastolic blood pressure, *SBP* systolic blood pressure.

### Primary outcome

**Figure 1:**
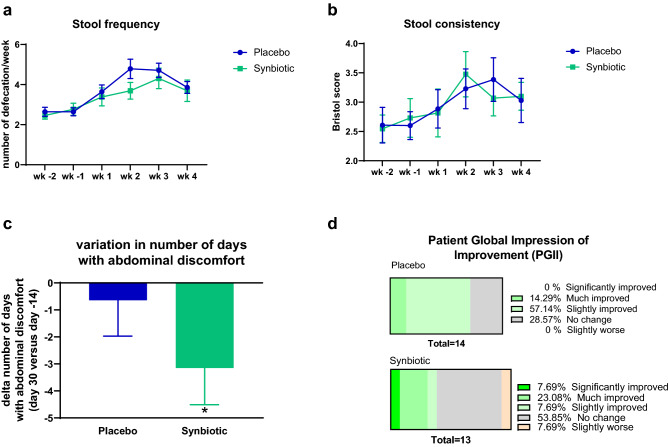
Transit characteristics in middle-aged adults receiving placebo or synbiotic for 30 days. Values are means ± SEM except for **d** (placebo: n = 14, synbiotic: n = 13). Mixed model ANOVA were performed to compare evolution of stool frequency and consistency (time effect: *p < 0.05). Matched-pairs Wilcoxon signed-rank test was performed for the number of days with abdominal discomfort to compare changes from baseline (*p < 0.05). *wk* week (wk-2: d-13 to d-7; wk -1: d-6 to d0; wk 1: d1 to d7; wk 2: d8 to d14; wk 3: d15 to d21; wk 4: d22 to d28).

Middle-aged subjects had experienced transit disorders for 17.8 ± 15.4 years on average and in the previous 2 weeks they had experienced pain on average on 2.9 ± 4.2 days. This pain was associated with feeling bloated in 55.6% of subjects and gas in 51.9% of them but never with diarrhea or liquid stools. Nearly one in five subjects (18.5%) had already taken treatments for transit, essentially laxatives of some sort. The average number of daily bowel movements observed in the reference period before taking the products under study was 0.4 ± 0.1 per day, that is, 2.6 ± 0.5 per week whereas stool consistency was 2.6 ± 0.9. After intervention, two of the Rome III diagnostic criteria for constipation were improved—stool frequency and consistency—over time whatever the treatments (placebo or synbiotic; Fig. 1a,b). In addition, the number of days with abdominal discomfort decreased in both groups but this effect was significant only after the treatment with the synbiotic (Fig. 1c). Symptoms accompanying abdominal pain or discomfort such as diarrhea, bloating or gas were not different between the treatments (Supplementary Table S1). 31% of the subjects in the synbiotic group, versus 14% in the placebo group, reported a significantly or much improved perception of change about the transit (according to the PGI-I questionnaire) (Fig. 1d).

### Secondary outcomes

**Figure 2:**
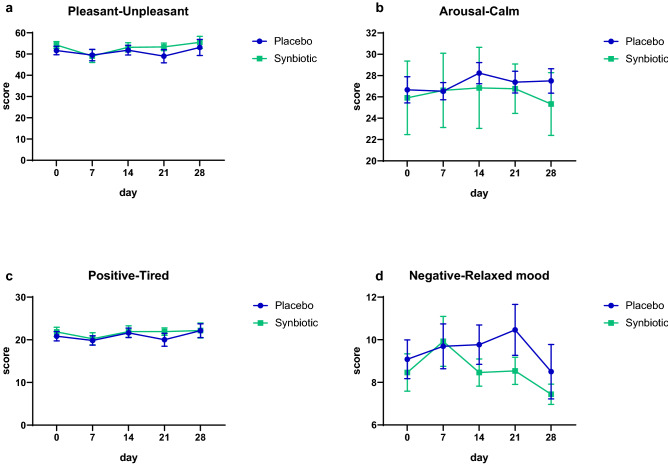
Mood subscales determined according to the Brief Mood Introspection Scale (BMIS) in middle-aged adults receiving placebo or synbiotic for 30 days. Values are means ± SEM (placebo: n = 14, synbiotic: n = 13; p > 0.05; mixed model ANOVA).

The change of habitual diet determined through a survey (established according to the recommendations of the French National Nutrition and Health Program (PNNS)) was not different between the groups except for sweetened foods (Supplementary Table S2). Indeed, we observed that the evolution in sweetened food intake was different between groups (30% of subjects consumed more sweetened food after 30 days of synbiotic supplementation whereas 21% of subject decreased this intake after placebo treatment). Of note, there was no difference in the evolution of water intake between groups. For the whole population, the Mental Component Score (MCS) was 47.4 ± 9.4 and the Physical Component Score (PCS) was 52.2 ± 6.5; both determined according the SF12 questionnaire. There was no significant difference between treatments regarding these scores (Table 2). In addition, mood tests did not show any significant general improvement whatever the items of the Brief Mood Introspection Scale (BMIS) or the groups considered (Supplementary Fig. S1). Figure 2 shows that there was no significant improvement in the four mood subscales over time whatever the treatments considered. However, it is worth noting that the scores for the Pleasant-Unpleasant dimension and Positived-Tired were higher in the synbiotic group whereas the scores for Arousal-Calm dimension and Negative-Relaxed mood dimension were lower in the synbiotic group compared to placebo group (except after 1 week of treatment where the scores were similar in both groups) (Fig. 2).

**Table 2:** Mental and physical wellbeing according to Short-Form 12-item (SF-12) questionnaire in middle-aged adults receiving placebo or synbiotic for 30 days.

	Placebo		Synbiotic	
	Baseline	Day 30	Baseline	Day 30
Mental Component Score (MCS)	45 ± 10.8	46.8 ± 11.3	50 ± 7.2	48.4 ± 5.8
Physical Component Score (PCS)	51.5 ± 7.9	50.6 ± 6.1	52.9 ± 5	54.1 ± 4.2
Physical function	87.5 ± 23.5	89.3 ± 18.9	94.2 ± 15	94.2 ± 11
Role physical	85.7 ± 18.9	84.8 ± 18.5	92.3 ± 10.9	95.2 ± 9.6
Bodily pain	78.6 ± 23.7	83.9 ± 23.2	90.4 ± 12.7	94.2 ± 11
General health	65.4 ± 10.6	62.9 ± 15.2	66.9 ± 13.6	68.1 ± 15.8
Vitality	33.9 ± 21	28.6 ± 25.7	46.2 ± 13.9	38.5 ± 21.9
Social function	89.3 ± 16.2	83.9 ± 23.2	90.4 ± 12.7	90.4 ± 12.7
Role emotional	75.9 ± 19.3	84.8 ± 18.5	92.3 ± 10.9	90.4 ± 11.6
Mental health	66.1 ± 23.2	66.1 ± 23.2	74 ± 15.7	73.1 ± 13.4

Values are means ± SD (placebo: n = 14, synbiotic: n = 13). Matched-pairs Wilcoxon signed-rank tests were performed to compare changes from baseline (within-group variations; p > 0.05). Between-groups variations were analyzed by Mann–Whitney U-tests (p > 0.05).

**Figure 3:**
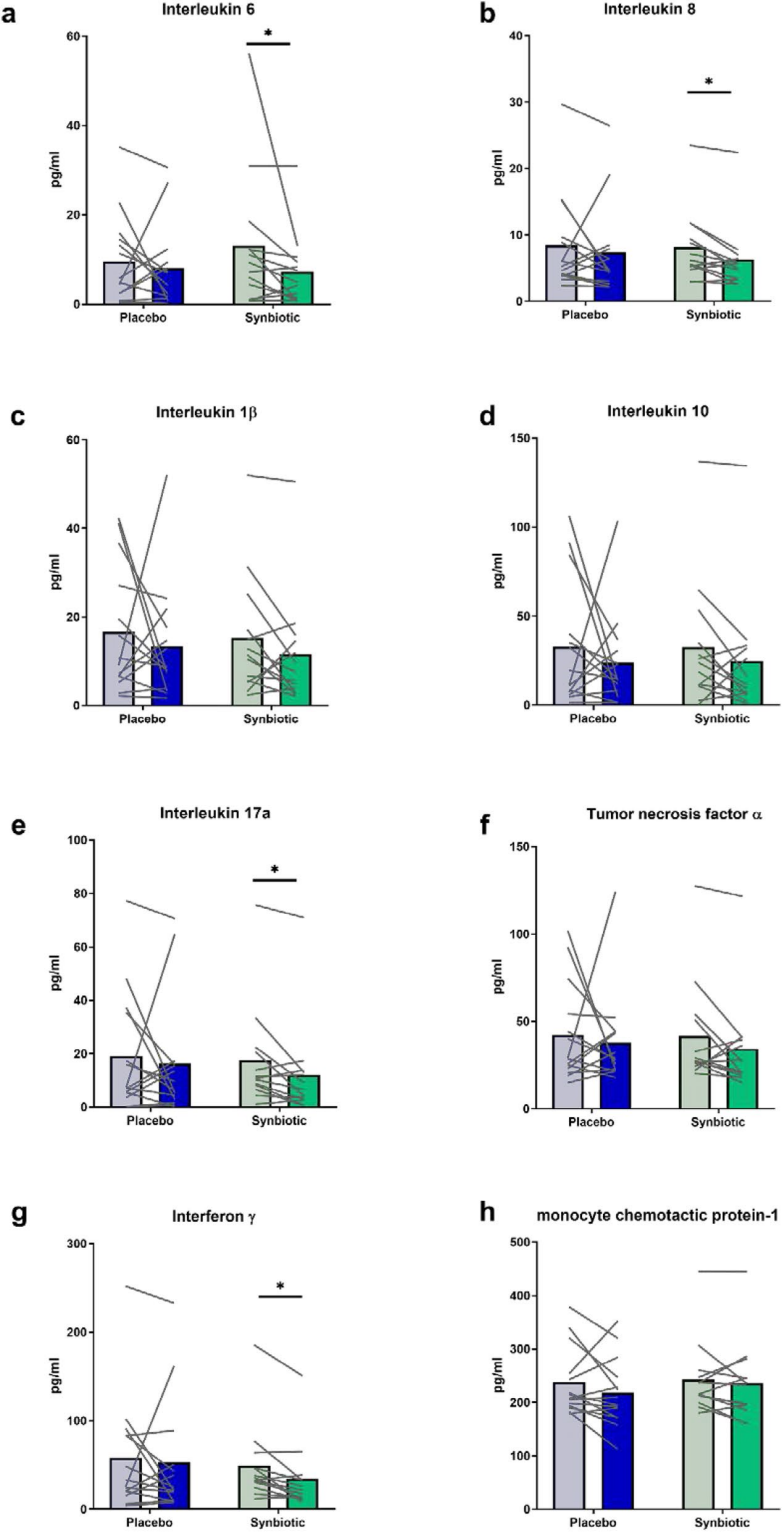
Plasma inflammatory markers in middle-aged adults at baseline (light color) or after receiving placebo or synbiotic for 30 days (dark color). Individual values and means are presented (placebo: n = 14, synbiotic: n = 13). Matched-pairs Wilcoxon signed-rank tests were performed to compare changes from baseline (within-group variations; *p < 0.05). Between-groups variations were analyzed by Mann–Whitney U-tests (p > 0.05).

Analysis of plasma inflammatory markers revealed that the synbiotic intervention impacted systemic inflammation. Indeed, the levels of IL-6, IL-8, IL-17a and IFNγ were significantly lower 30 days after synbiotic treatment as compared to the basal values (Fig. 3). This effect was not present in the placebo group and seemed to be independent of the gut permeability as suggested by the lack of significant effect on fecal albumine or plasma intestinal fatty acid binding protein (iFABP) whatever the treatment considered (Supplementary Fig. S2).

**Figure 4:**
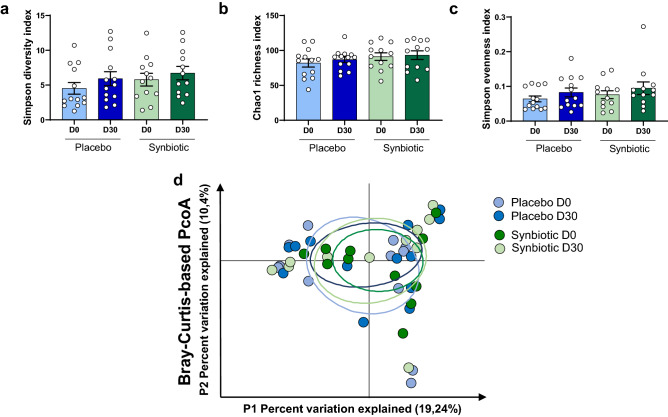
Alpha-diversity indexes (**a**–**c**) and principal coordinates analysis of the β-diversity index, Bray–Curtis (**d**) of the gut microbiota in middle-aged adults at baseline or after receiving placebo (n = 14) or synbiotic (n = 13) for 30 days. Data are mean ± SD. For α-diversity indexes, matched-pairs Wilcoxon signed-rank tests were performed to compare changes from baseline (within-group variations; p > 0.05). For β-diversity index, a Monte-Carle rank test was performed. Between-groups variations were analyzed by Mann–Whitney U-tests (p > 0.05).

As shown in Fig. 4, there were no statistically significant differences in the alpha and beta diversities plots after 30 days of placebo or synbiotic. Coherently, the relative abundance of the most abundant phyla, family and genera were unchanged by the treatments (data not shown).

## Discussion

It has already been suggested that constipation associated with advanced age is an adequate condition for observing an improvement of intestinal transit time after probiotic supplementation^19^. A meta-analysis conducted in 2017 and updated in 2019^24,25^ has concluded that synbiotics have a positive effect on bowel movement frequency and stool consistency. It should be noted that all studies using FOS or *B. animalis* in the synbiotic combinations were performed in adults below 40 years. Therefore, the primary objective of our randomized, double-blind, controlled clinical trial was to test the influence of a synbiotic combination composed of FOS and *Bifidobacterium animalis* subsp. *lactis*. in older healthy volunteers with infrequent bowel movements (but not severe constipation). The selected probiotic and prebiotic have been often tested alone showing an improvement in intestinal transit especially in the elderly^26,27^. Consistent with a more recent study performed in subjects of 45 years old treated with *B. animalis* subsp. *lactis* and inulin^28^, the present study reports that stool frequency and consistency improved over time whatever the treatment (placebo or synbiotic) in subjects with a mean age of 58 years. Importantly, the number of days with abdominal discomfort decreased only in the synbiotic group and 30% of the subjects perceived significant improvement of their gut transit time after the synbiotic treatment.

Dysbiosis in functional constipation (Rome III criteria) were recently reviewed^25^. In particular, Khalif et al. demonstrated that patients with functional constipation (mean age 42 years) had a reduced level of *Bifidobacterium, Lactobacillus, Bacteroides,* and *Clostridium* species and an increased level of *Enterobacteriaceae*, such as *Escherichia coli*, as well as *Staphylococcus aureus* and fungi^29^. This study and other published data therefore suggest that constipation is associated with gut dysbiosis, and that gut motility can be managed by modulation of gut microbiota^30–32^. Others reported that relief of constipation by laxatives tend to normalize and restore microbiota composition, thus suggesting that dysbiosis is a consequence, rather than a cause of constipation^29^. In the current study, we did not find any changes in the relative abundance of the most frequent phyla, family and genera in this middle-aged population who met Rome III criteria for constipation in the synbiotic nor placebo treated groups. This suggests that the gut microbiota composition was not relevant in the improvement of the stool frequency and consistency or abdominal discomfort pinpointed in the present study. This lack of association was already reported in a randomized, placebo-controlled trial testing the effects of inulin on stool frequency, gut microbiota composition and quality of life in middle-aged to older populations^21^. This study demonstrated that inulin (10 g/day) improved stool frequency and consistency without significant changes in gut microbiota composition after 5 weeks of treatment. We cannot exclude however that bacterial species below the limit of detection of 16S rDNA sequencing may be affected by the synbiotic and may contribute to the observed benefits.

A decline in the quality of life related to constipation and bowel-related symptoms has been reported and reached the same extent as some chronic conditions like diabetes^4,33^. Therefore, we assessed quality of life using the SF12 survey. This questionnaire allowed us to evaluate the level of eight health concepts including components of both physical and mental health. Synbiotic supplementation or placebo treatment did not change any of these eight categories meaning that both interventions did not improve neither physical health nor mental health of participants. In line with this result, although four different dimensions calculated from BMIS questionnaire tended to be improved by the synbiotic treatment, none of those revealed significant impact of dietary supplementations on subject mood.

Previous studies have demonstrated that older individuals with higher levels of inflammatory markers are more likely to develop a variety of late-life diseases, accompanied by a higher hospitalization rate and all-cause mortality rate ^13^. Of particular interest, one study, consistent with previous investigations, demonstrates that subclinical elevations of inflammatory markers, in particular IL-6, are associated to the development of diabetes in middle-aged adults^10^. Several studies have suggested the potential contribution of microbiota-driven therapies to improve inflammatory states in inflammatory bowel disease, obesity and type 2 diabetes^9,34–37^. In addition, other studies also showed a benefit of microbiota-driven therapies, including synbiotic in elderly individuals (> 60 years), in the context of inflammatory disorders^38,39^. Although previous animal studies have demonstrated a benefit of microbiota-driven therapies to decrease low grade chronic inflammation in aged individuals, it remains controversial in human interventions^9,23^. A systematic review conducted in 2019 investigated the effect of microbiota-driven therapies in randomized controlled trials on inflammatory markers in elderly individuals. The authors concluded that after treatment, no differences were observed between microbiota-driven therapy group and placebo group in the levels of key proinflammatory cytokines such as TNF-α, IL-1β, IL-6 and IL-8^9^. In the present study, we observed that the synbiotic combination decreased the key cytokine IL-6 among other proinflammatory markers (IL-8, IL17a and IFNγ) after only 30 days of treatment in healthy middle-aged volunteers without any suspicion of type 2 diabetes mellitus and cardiovascular diseases. This anti-inflammatory effect seemed to be independent of the gut barrier integrity as suggested by the lack of significant effect on fecal albumine or plasma iFABP^40^. Recent papers propose that intestinal bacterial metabolites modulate inflammation and gene expression, notably in the liver (for review, see^35^). An important clinical trial in in nonalcoholic fatty liver disease (NAFLD) patients consuming 4 g FOS twice daily combined with *B. animalis* subsp. *lactis* BB-12 (INSYTE study) has demonstrated that changes with this synbiotic in specific microbes known to be involved in the regulation of the inflammatory tone occur without clinically significant effects on the liver. This supports a need to evaluate the effect of synbiotic treatment on inflammatory/immune-related parameters in NAFLD^41^. Along these lines, another study showed that the consumption of a yogurt containing *B. animalis* subsp. *lactis* produced intestinal bacterial metabolites that contribute to suppression of inflammatory cytokines produced by macrophages. One of the anti-inflammatory metabolites in the fecal extracts was likely a polyamine^42^.

Importantly, IL-17 plays a crucial role in sustaining chronic inflammation and its over-expression has been found in a number of inflammatory disorders, including inflammatory bowel disease^43^. Ligation of IL-17 with its receptor may induce IL-6 and IL-8 production through mitogen-activated protein kinase pathways, thus favoring the recruitment of neutrophils at sites of inflammation, and triggers T cell proliferation and upregulation of a number of pro-inflammatory molecules.

We have identified several limitations to our study. First, the treatment lasted 30 days. De facto, the effect of a longer term synbiotic supplementation on (gastrointestinal) wellness and inflammation is unknown. Secondly, a study with a higher number of participants and a cross-over design would increase the statistical power of the analyses. Thirdly, knowing that gender may impact outcomes related to constipation or the prevalence and severity of constipation in the elderly, this parameter should be tested as possible effect modifiers by choosing for example to enroll higher number of subjects with control, power and randomization for gender in a future cross-over study^44^. Research is needed on gender and age differences in the symptoms of constipation, and how covariates impact the prevalence and severity of constipation in the elderly. Fourthly, the gastrointestinal symptoms such as discomfort, nausea, flatulence, cramp, burp, bloating, rumbling and reflux were not investigated on visual analogue scales (VAS). Nevertheless, we can conclude a combination of *B. animalis* subsp. *lactis* with the well-known prebiotic FOS could be a promising synbiotic strategy to decrease inflammatory status with improvement of gut disorders in middle-aged people that experienced episodes of constipation. In line with the current literature, we propose that the early treatment of systemic inflammation through microbiota-driven therapies such as synbiotics may improve prognostic of chronic inflammatory-dependent disorders such as inflammatory bowel disease, type 2 diabetes mellitus and cardiovascular diseases^45^.

## Methods

### Power analysis and sample size

The sample size assumes a difference of 1 in the average change in stool frequency per week between the synbiotic group and the placebo group. For the randomized, placebo-controlled study design and using the outcome measures described in the study protocol, the calculation performed (with Nquery Advisor software version 7.0; https://www.statsols.com/nquery), with a standard deviation of 1, an increase of 0.5 stools per week in the placebo group and 1.5 stools in the synbiotic groups at risk alpha = 0.05, with a power of 80 and in a unilateral situation, gives a number of subjects to introduce of 13 per arm (30 subjects in total to take into account the loss of sight and the non-exploitable files).

### Clinical study

This prospective, randomized, double-blind, placebo-controlled study was designed according to the CONSORT 2010 guidelines and was conducted at the CEN Nutriment, a Contract Research Organization in France (Dijon) from March 2017 to March 2018. The study was approved by the Comité de Protection des Personnes (Dijon, France; Approval Number: 2016-A00970-51) prior to implementation. The trial study was listed on the NIH ClinicalTrials.gov website (NCT04283266; 25/02/2020). No changes were made to this trial after recruitment of the participants commenced. The author ensure that the study has been carried out in accordance with The Code of Ethics of the World Medical Association and followed the ethical guidelines set out in the Declaration of Helsinki. All participants provided written informed consent in compliance with the European law 2001/20/CE guidelines.

The allocation sequence to either placebo or study product was based on random sequence generated using MS EXCEL (simple randomization). The products were distributed to subjects in accordance with the randomization list. The randomization key, indicating to which products the batches given to patients corresponded to, was kept in a sealed envelope. The coordinator and nurse enrolled participants and assigned them to placebo or synbiotic (computer-generated randomized numbers). Participants, care providers, researchers involved in data analyses were blinded to which arm participants were assigned. All information collected was kept in a secured area and sent for statistical analyses with a study number and without participant identifiers.

**Figure 5:**
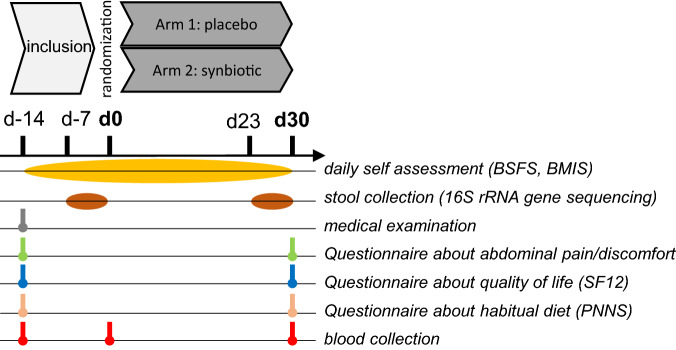
Overview of the clinical study. *BMIS* Brief Mood Introspection Scale, *BSFS* Bristol Stool Form Scale; *SF12* 12-item short form survey measuring the eight health domains for adults (providing psychometrically-based physical component summary and mental component summary scores), *PNNS* French National Nutrition and Health Program.

An overview of the study design is shown in Fig. 5. Forty subjects were recruited. Among them, 13 subjects were excluded from the analysis because they did not meet the inclusion criteria. Twenty-seven received allocated intervention and completed the study (Supplementary Fig. S3). The inclusion criteria and exclusion criteria were given in “Supplementary Methods S1”. Of note, knowing that physical exercise may affect gut transit^46^, the volunteers were instructed not to change their physical activity habits during the study.

The primary endpoint of this trial is to evaluate the effect of synbiotic supplementation on intestinal transit of subjects. The secondary endpoints are to evaluate changes in stool appearance, quality of life and mood, relief and satisfaction of participants, changes in food consumption (survey with items according to recommendations of French National Nutrition and Health Program^47^) and changes in low-grade chronic inflammation and markers of gut function.

### Intervention

The subjects received daily two bags of either synbiotic (*Bifidobaterium animalis lactis Vesalius 002* (LMG P-28149) 5.10^9^ bacteria/bag + fructo-oligosaccharides (FOS, ACTILIGHT, 4.95 g/bag) or placebo (maltodextrin 60%, sucrose 40%; 5 g/bag) for 5 days, then they received daily 1 bag for the next 25 days. As already described for other strains of *Bifidobaterium animalis lactis*^48^, this strain is resistant to the stomach or small intestinal environment (data not shown). Bags were prepared by Vesale Pharma (Belgium) and were strictly identical in appearance. Placebo powder contained only excipients (maltodextrin 60%–saccharose 40%). The white powder was also similar in appearance, was odorless and had similar sweet taste for both packaging. Subjects received 50 bags in total but only 35 were required to complete the study; unused bags were returned to measure compliance (calculation: (50 − number of bags that were returned) × 100/35). The sachets were to be dissolved in 200 ml of water at room temperature to be taken before breakfast.

### Transit characteristics

The stool frequency and consistency were investigated through daily self-assessment using the Bristol Stool Form Scale (BSFS)^49^, a simple tool for estimating intestinal transit time, from 2 weeks before the enrolment visit (run-in period) until the end of the intervention. The BSFS classifies stools into seven categories, including type 1, separate hard lumps, like nuts; type 2, sausage-shaped, but lumpy; type 3, like a sausage but with cracks on the surface; type 4, like a sausage or snake, smooth and soft; type 5, soft blobs with clear-cut edges; type 6, fluffy pieces with ragged edges, a mushy stool; type 7, watery, no solid pieces^50^. These types are categorized into slow transit (types 1 and 2), normal transit (types 3–5), and fast transit (types 6 and 7).

A questionnaire was submitted at baseline (d-14) and after intervention (d30) in order to evaluate the number of days with abdominal pain and intestinal discomfort evolution (accompanied or not with symptoms of diarrhea, bloating or gas). Patient Global Impression of Improvement (PGI-I) survey was also submitted at the end of the treatment (day 30); the PGI-I is a 1-item questionnaires asking to rate the perceived change in his/her condition in response to therapy at endpoint^51^.

### Mood assessment

Mood alterations was investigated through daily self-assessment using the Brief Mood Introspection Scale (BMIS), consisting of 16 mood adjectives (Lively, Drowsy, Happy, Grouchy, Sad, Peppy, Tired, Nervous, Caring, Calm, Content, Loving, Gloomy, fed up, Jittery, Active)^52^. Subjects were asked to circle the phrases describing their present mood (with XX = definitely do not feel; X = do not feel; V = slightly feel; VV = definitely feel on the diverse adjectives). The BMIS was scored for Pleasant-Unpleasant Mood, Arousal-Calm Mood, Positive-Tired Mood and Negative-Relaxed Mood according to the Mayer’s method^53^.

### Quality of life assessment

Mental and physical wellbeing were assessed before (d-14) and after intervention (d30) through the Short-Form 12-item (SF-12) questionnaire consisting of 12 questions relating to: physical health problems, bodily pain, general health perceptions, vitality (energy/fatigue), social functioning, role limitations and general mental health (psychological distress and psychological well-being)^54,55^. This instrument yields two summary scores: a Mental Component Score (MCS), and a Physical Component Score (PCS). The SF12 takes 2 min to administer and has been validated for use with elderly people*.*

### Plasma analysis

The blood samples collected before (d-14) and after intervention (d30) were immediately centrifuged and plasma was transferred and kept at − 20 °C until analysis. Biochemical parameters including alanine aminotransferase (ALAT) and aspartate aminotransferase (ASAT) concentrations were measured using standard laboratory techniques. Blood for peptide analysis was immediately transferred into specific tube (BD P800 Blood Collection System (BD Biosciences, CA, USA). Plasma obtained for inflammatory markers and peptides were transferred at − 80 °C before analysis. Plasma cytokines (interleukin (IL)-1β, IL-6, IL-8, IL-10, IL-17a, monocyte chemotactic protein-1 (MCP-1), interferon (IFN) γ and tumor necrosis factor (TNF) α) and plasma peptides (pancreatic polypeptide (PP), glucose-dependent insulinotropic peptide (GIP), leptin, ghrelin, insulin) were determined in duplicate by multiplex immunoassays (Millipore, Belgium) and measured using LUMINEX xMAP technology (Biorad, Nazareth, Belgium) following the manufacturer’s instructions*.*

### Gut microbiota analyses

Stool samples were collected at baseline and at the end of the 30-days of intervention (one fecal sample collected and stored at − 20 °C within 7 days before day 0 and before day 30) and transferred to − 80 °C for the analysis of the gut microbiota composition. Genomic DNA was extracted from faeces using a PSP spin stool plus DNA kit (Stratec biomolecular, Berlin, Germany), according to the manufacturer’s instructions. 16S rDNA profiling, targeting V1–V3 hypervariable region and sequenced on Illumina MiSeq were performed as described previously^56^.

### Statistical analysis

Data are expressed as mean ± SD for tables and mean ± SEM for figures. Between group differences were analyzed by Fisher or Chi Square tests for categorical variables or Mann–Whitney test for continuous variables. Within group analyses were evaluated using a Wilcoxon paired test (from baseline to 30 days of intervention). Mixed model ANOVA by Sidak’s multiple comparisons test were performed to compare effects over time. A significance level of p < 0.05 was used for all the analyses. For gut microbiota analysis, p-values of within group comparisons were corrected to control for the false discovery rate (FDR) for multiple tests according to the Benjamini and Hochberg procedure and a significance level of q < 0.1 was used. All analyses were conducted with, Graphpad Prism software version 8 (San Diego, USA; www.graphpad.com) except for the gut microbiota analysis where we used R version 3.5.2.

## Supplementary Information

Supplementary Information.

## Abbreviations

ALAT: Alanine aminotransferase
ASAT: Aspartate aminotransferase
BMI: Body mass index
BMIS: Brief Mood Introspection Scale
BSFS: Bristol Stool Form Scale
DBP: Diastolic blood pressure
FOS: Fructo-oligosaccharides
iFABP: Intestinal fatty acid binding protein
IL: Interleukin
IFNγ: Interferon-gamma
MCP-1: Monocyte chemotactic protein-1
MCS: Mental Component Score
NAFLD: Nonalcoholic fatty liver disease
PGI-I: Patient Global Impression of Improvement survey
PCS: Physical Component Score
SBP: Systolic blood pressure
SF12: 12-Item short form survey
VAS: Visual analogue scales
